# Perceptual and Acoustic Analysis of Speech in Spinocerebellar ataxia Type 1

**DOI:** 10.1007/s12311-023-01513-9

**Published:** 2023-01-12

**Authors:** Teije van Prooije, Simone Knuijt, Judith Oostveen, Kirsten Kapteijns, Adam P. Vogel, Bart van de Warrenburg

**Affiliations:** 1 Department of Neurology, Donders Institute for Brain, Cognition and Behaviour, Radboud University Medical Center, Nijmegen, Netherlands; 2 Department of Rehabilitation, Donders Institute for Brain, Cognition, and Behaviour, Radboud University Medical Center, Nijmegen, Netherlands; 3 Centre for Neuroscience of Speech, The University of Melbourne, Melbourne, Australia; 4 Translational Genomics of Neurodegenerative Diseases, Hertie-Institute for Clinical Brain Research and Center of Neurology, University of Tübingen, Tubingen, Germany; 5 Redenlab Inc., Melbourne, Australia

**Keywords:** Spinocerebellar ataxia type 1, Speech, Dysarthria

## Abstract

This study characterizes the speech phenotype of spinocerebellar ataxia type 1 (SCA1) using both perceptual and objective acoustic analysis of speech in a cohort of SCA1 patients. Twenty-seven symptomatic SCA1 patients in various disease stages (SARA score range: 3–32 points) and 18 sex and age matched healthy controls underwent a clinical assessment addressing ataxia severity, non-ataxia signs, cognitive functioning, and speech. Speech samples were perceptually rated by trained speech therapists, and acoustic metrics representing speech timing, vocal control, and voice quality were extracted. Perceptual analysis revealed reduced intelligibility and naturalness in speech samples of SCA1 patients. Acoustically, SCA1 patients presented with slower speech rate and diadochokinetic rate as well as longer syllable duration compared to healthy controls. No distinct abnormalities in voice quality in the acoustic analysis were detected at group level. Both the affected perceptual and acoustic variables correlated with ataxia severity. Longitudinal assessment of speech is needed to place changes in speech in the context of disease progression and potential response to treatment.

**Supplementary Information:**

The online version contains supplementary material available at 10.1007/s12311-023-01513-9.

## Introduction

Spinocerebellar ataxia type 1 (SCA1) is one of the most progressive subtypes of autosomal dominant SCAs [1]. The genetic cause of SCA1 is an expanded CAG-repeat in the *ATXN1* gene (chromosome 6p22.3), resulting in a mutant ataxin-1 protein containing expanded polyglutamine stretches [2]. This disease mechanism is shared with other polyglutamine diseases, such as SCA2, SCA3, SCA6, and Huntington’s disease (HD)[3]. Recently, significant steps towards potential genetic, disease-modifying treatments for polyglutamine diseases have been made, but objective (bio) markers to track disease progression and sensitive yet meaningful clinical endpoints for these disease-modifying trials are lacking [4].

Progressive dysarthria is a devastating feature of most ataxias, including SCA1. Perceptual and acoustic analysis of cerebellar dysarthria in other genetic ataxias previously revealed speech phenotypes with deficits in the domains of voice quality, speech, and articulatory timing and control [5]. Ataxic dysarthria is characterized by slow and irregular speech with increased pauses and imprecise consonants. In general, articulatory deficits are the most prominent feature of ataxic dysarthria. A study in multiple SCA genotypes suggested that in SCA1, voice quality might be more affected than articulation [6].

A role for speech impairment as a potential objective marker to track disease progression has been previously explored in several genetic ataxias and polyglutamine diseases presenting with dysarthria, such as Friedreich ataxia (FRDA) [7, 8], SCA2 [9], autosomal recessive spastic ataxia of Charlevoix-Saguenay (ARSACS) [10], POLG-associated ataxia [11], and HD [12–14]. Digital speech testing has identified deficits associated with disease progression and ataxia severity in SCA2 [9], as well as longitudinal changes in speech after 2 years of follow-up in FRDA [15]. Further, subtle acoustic speech deficits relating to speech-timing appeared detectable in pre-ataxic SCA2 mutation carriers [9] and in early symptomatic HD patients [12, 13], suggesting a promising role for acoustic measures of speech as a marker for disease onset and progression.

The speech phenotype in SCA1 has not been fully characterized, and acoustic speech markers have not been examined. Detailed characterization of speech deficits in SCA1, covering all disease stages and speech subsystems, is needed to explore the role of speech as a focused motor marker for tracking disease progression and as a potential clinical endpoint in future disease-modifying trials. The objective of this study was to cross-sectionally map the most distinctive features of cerebellar dysarthria in SCA1 in various disease stages and correlate clinician reported and performance outcomes of speech with relevant disease metrics.

## Methods

### Participants

Twenty-seven symptomatic SCA1 patients and eighteen healthy controls matched for sex and age participated in this study, which is part of a longitudinal Dutch natural history and biomarker discovery study in SCA1. All participants were native Dutch speakers. The Scale for Assessment and Rating of Ataxia score (SARA score) was used to determine clinical severity of ataxia [16]. SCA1 mutation carriers were classified as symptomatic if their SARA score was ≥3. The clinical Inventory of Non-Ataxia Signs (INAS) [17] was used to assess the presence of non-ataxia signs. Cognitive functioning was screened using the Montreal Cognitive Assessment (MoCA) [18].

### Speech Assessment

Speech and voice samples were recorded in a quiet room, with a head mounted condenser microphone (AKG c520, Austria), coupled with an external audio interface (Rubix 24, Roland, Japan) and a laptop using the Redenlab® Desktop software. All participants completed a set of seven speech tasks in one sitting. Tasks fit along a continuum of motor and cognitive complexity [19, 20] and have known stability in repeated application [21]. All tasks were administered and completed in Dutch.

1. Paragraph reading task (Dutch translation of “The North Wind and the Sun”).
2. Produce the days of the week (twice) (automated task).
3. Produce a sustained vowel: (/ɑ:/) for as long as possible on one breath (twice).
4. Produce a clear sustained vowel : (/ɑ:/) for 5 seconds.
5. Sequential motion rate task, producing the “Pa-Ta-Ka” syllables as quickly and clearly as possible for 10 seconds (twice).
6. Unprepared monologue for 1–2 min.
7. Picture description task, describing elements on a picture to elicit semi-spontaneous speech.

### Clinician-Reported Outcomes

Two trained speech therapists with expertise in ataxic dysarthria rated speech samples for intelligibility, naturalness, and dysarthria severity (in monologue only). Both raters listened to and rated the recordings of one participant separately before reaching a consensus for every participant. Intelligibility and naturalness were rated using direct magnitude estimation (DME) [22], where participants were compared to an established anchor (mild dysarthria). The anchor was given a value of 100. If the speaker was twice as intelligible or natural as the anchor, the score was 200. If the speaker was half as intelligible or natural as the anchor, the score was 50. Overall dysarthria severity was scored on the 6-point Radboud Dysarthria Assessment scale for dysarthria severity (0–5: 0 = no dysarthria, 1 = minimal dysarthria, 2 = mild dysarthria, 3 = mild/severe dysarthria, 4 = severe dysarthria, 5 = very severe dysarthria/anarthria) [23]. Both raters were blinded for disease status (SCA1 patient/healthy control). Speech disturbance was also perceptually rated between 0 (normal) and 6 (speech unintelligible) by a neurologist, as one of the items of the SARA assessment.

### Performance Outcomes

Speech samples were analyzed acoustically using previously described [8, 21, 24] purpose-built scripts run through Matlab or PRAAT software [25]. Acoustic variables representing aspects of speech timing were extracted from the reading task, the automated task, the monologue task, picture description task, and sequential motion rate task (speech rate and diadochokinetic rate, syllable duration and variability, pause length, pause variability, and percent of pauses in monologue). Acoustic variables representing vocal quality (recurrence period density entropy, harmonics to noise ratio) and control (fundamental frequency coefficient of variation) were extracted from the vowel samples.

### Statistical Analysis

Mean and SD values were calculated for both acoustic and perceptual speech variables and all clinical characteristics. Means of perceptual speech variables were compared between SCA1 patients and healthy controls using non-parametric tests (Mann-Whitney *U* test). The means of acoustic speech variables were compared between the group with SCA1 patients and healthy controls using parametric tests if data were normally distributed. Some acoustic variables did not present with a normal distribution and were transformed using natural log transformation. For all acoustic speech variables, Cohen’s *d* was calculated as universal effect size.

For perceptual speech variables (*α* = 0.02 (0.05/3)) and acoustic variables representing speech timing (*α* = 0.007 (0.05/7)) or voice quality and control (*α* = 0.008 (0.05/6)), statistically significant group differences were adjusted for multiple testing using Bonferroni method.

The relationship between perceptual and acoustic speech variables and disease severity was explored by calculating (Spearman rho) correlation coefficients between acoustic or perceptual speech variables that showed statistically significant between-group differences and SARA scores. To assess the relationship between dysarthria and the severity of (limb and gait) ataxia, correlation coefficients were also calculated between speech variables and SARA scores corrected for speech impairment (SARA_adj_ = SARA total − SARA speech). We also assessed a possible relationship between acoustic and perceptual speech variables and disease duration, CAG repeat length (expanded allele), and cognitive functioning using adjusted total MoCA scores (MoCA_adj_ = MoCA total scores − MoCA visuospatial items). Total MoCA scores were adjusted because several SCA1 patients were not able to complete visuospatial tasks due to severe limb ataxia (*n* = 5). One healthy control was excluded in the correlation analysis with cognitive function because performance was affected by severe dyscalculia.

To explore the potential of acoustic speech analysis to detect subtle acoustic changes in SCA1 patients with perceptually absent or minimal dysarthria, a subgroup analysis was performed for those acoustic measures that showed significant between-group differences and correlated well with disease severity. SCA1 patients were divided into two groups based on the perceptually perceived dysarthria severity scores: no/minimal dysarthria group (*n* = 8) and mild/severe dysarthria group (*n* = 19). Means of the selected acoustic variables were compared between the no/minimal dysarthria group and healthy controls using (two-tailed) *T*-tests.

R-studio 1.1.463 was used for all statistical analysis.

## Results

### SARA Scores and Neurologist Rating of Speech

The mean SARA score for the 27 SCA1 patients was 14.4 points (range 3–32 points, SD ± 7.4). The SARA speech score (ranging between 0 and 6) rated by a clinician was on average 2.1 points (range 0 – 4, SD ±1.2) within this group. Two SCA1 patients presented with a SARA speech score of 0, indicating no dysarthria. Eight SCA1 patients had subtle dysarthria, as indicated by a SARA speech score of 1 (“suggestion of speech impairment”). All other SCA1 patients presented with mild (*n* = 7), moderate (*n* = 6), or severe (*n* = 4) dysarthria. All healthy controls scored 0 on the SARA speech score and a mean of 0.44 (± 0.59) points on SARA total score. See Table 1 for all participant characteristics.

**Table 1:** Participant characteristics (data are presented as mean ± SD)

	Healthy controls	SCA1 patients	*p*-value
No.	18	27	–
Sex (M/F)	9/9	15/12	*0.68*
Age (y)	48.6 ± 12.9	52.4 ± 13.1	*0.33*
*Range (y)*	*28.9–67.8*	*29.9–75.1*	–
Disease duration (y)	–	8.65 ± 6.1	–
*Range (y)*	–	*0–21*	–
Expanded allele length (CAG repeats)	–	45.2 ± 4.4	–
*Range (CAG repeats)*	–	*39–53*	–
Ataxia severity (SARA)	0.44 ± 0.59	14.4 ± 7.4	*< 0.001*
*Range (SARA)*	*0–2*	*3–32*	–
*SARA speech score*	*0*	*2.1* ± 1.2	*< 0.001*
*Range (SARA speech)*	*-*	*0–4*	–
Number of non-ataxia signs (INAS)	0.39 ± 0.7	4.4 ± 2.2	*< 0.001*
*Range (INAS)*	*0–2*	*1–8*	–
Montreal Cognitive Assessment (MoCA) scores	27.7 ± 1.5 (*n* = 17)	26.2 ± 2.1 (*n* = 20)	*0.01*
*Range (MoCA)*	*24–30* (*n* = 17)	*24–29 (n = 20)*	–

### Perceptual Speech Variables

Perceptual speech analysis revealed a mean of 2.2 points on the dysarthria severity score (± 1.4; range: 0–5) in the group of SCA1 patients. Four SCA1 patients (with total SARA scores ranging between 3 and 8 points) scored 0 points on the dysarthria severity score, thus indicating no dysarthria. Two out of these four patients also received 0 points on the SARA speech item. The other two patients (with total SARA scores of 5.5 and 8 points) scored 1 point on the SARA speech score. The overall correlation between dysarthria severity score and SARA speech was high (*ρ* = 0.86, *p*-value <0.0001) in the SCA1 group. One healthy control scored 1 point on the dysarthria severity score, resulting in a mean score of 0.11 points (± 0.3, range: 0–1) for this group (group difference, Cohen’s *d* = 2.03, *p*-value < 0.0001).

SCA1 patients scored significantly lower on direct magnitude estimation (DME) for both intelligibility (mean DME SCA1 patients: 96 (± 59.5), Cohen’s *d* = −2.19, *p*-value <0.0001) and naturalness (mean DME SCA1 patients: 102 (± 54.8), Cohen’s *d* = −2.17, *p*-value <0.0001) (see also Appendix 1a) compared to healthy controls. Four SCA1 patients received unremarkable DME scores for intelligibility and five SCA1 patients scored unremarkable DME scores for naturalness. All healthy controls received maximal DME scores for both naturalness and intelligibility.

### Acoustic Speech Variables

**Fig. 1:**
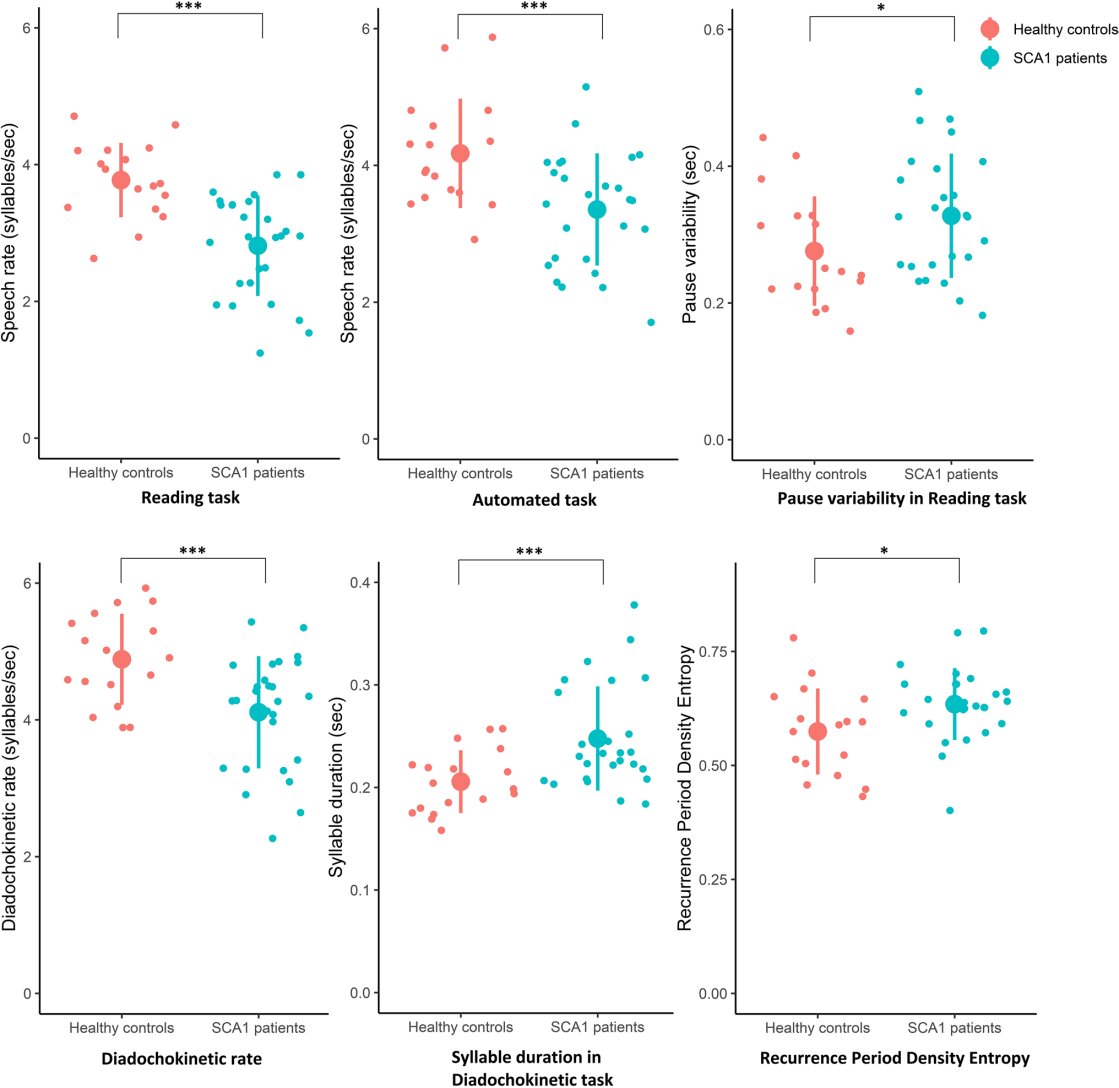
Group differences between SCA1 patients and healthy controls for acoustic variables representing aspects of speech timing and voice quality (individual data, mean ± SD are presented). ***p ≤ 0.002, *p-value < 0.05. For speech rate in automated task, one outlier (healthy control) was excluded because of a clear discrepancy with speech rate in reading task

Statistically significant between-group differences with large effect sizes were observed for speech rate *in reading task* (Cohen’s *d* = −1.48, *T*-value −5.03, *p*-value <0.0001), speech rate *in automated task* (Cohen’s *d* = −1.01, *T*-value −3.28, *p*-value = 0.002), diadochokinetic rate (Cohen’s *d* = −1.1, *T*-value −3.67, *p*-value <0.001), and syllable duration (Cohen’s *d* = 1.0, *T*-value 3.7, *p*-value <0.001). One additional acoustic variable representing timing of speech (variability in pause length *in reading passage*, *T*-value 2.5, *p*-value 0.02) and one variable representing an aspect of voice quality (recurrence period density entropy *in sustained vowel task*, *T*-value 2.2, *p*-value 0.03) were significantly different between groups with moderate effect sizes but failed to reach significance after adjustment for multiple comparisons (see Fig. 1). We did not observe statistically significant group differences for other acoustic variables reflecting voice quality (harmonics to noise ratio (*T*-value −1.9, *p*-value = 0.07) and vocal control in fundamental frequency coefficient of variation (*T*-value 1.43, *p*-value = 0.16 *in sustained vowel*, *T*-value 0.22, *p*-value 0.82 *in monologue*)). See Appendix 1b for mean, SD, effect size, and *T*-test results of all acoustic variables.

### Correlation with Disease Severity and Duration, CAG Repeat Length, and Cognitive Functioning

The perceptual variables DME intelligibility (*ρ* = −0.74, *p*-value <0.0001) and DME naturalness (*ρ* = −0.81, *p*-value <0.0001) and dysarthria severity score (*ρ* = 0.78, *p*-value <0.0001) all showed a strong significant correlation with SARA scores and similar effect sizes when correlated with adjusted SARA scores.

**Fig. 2:**
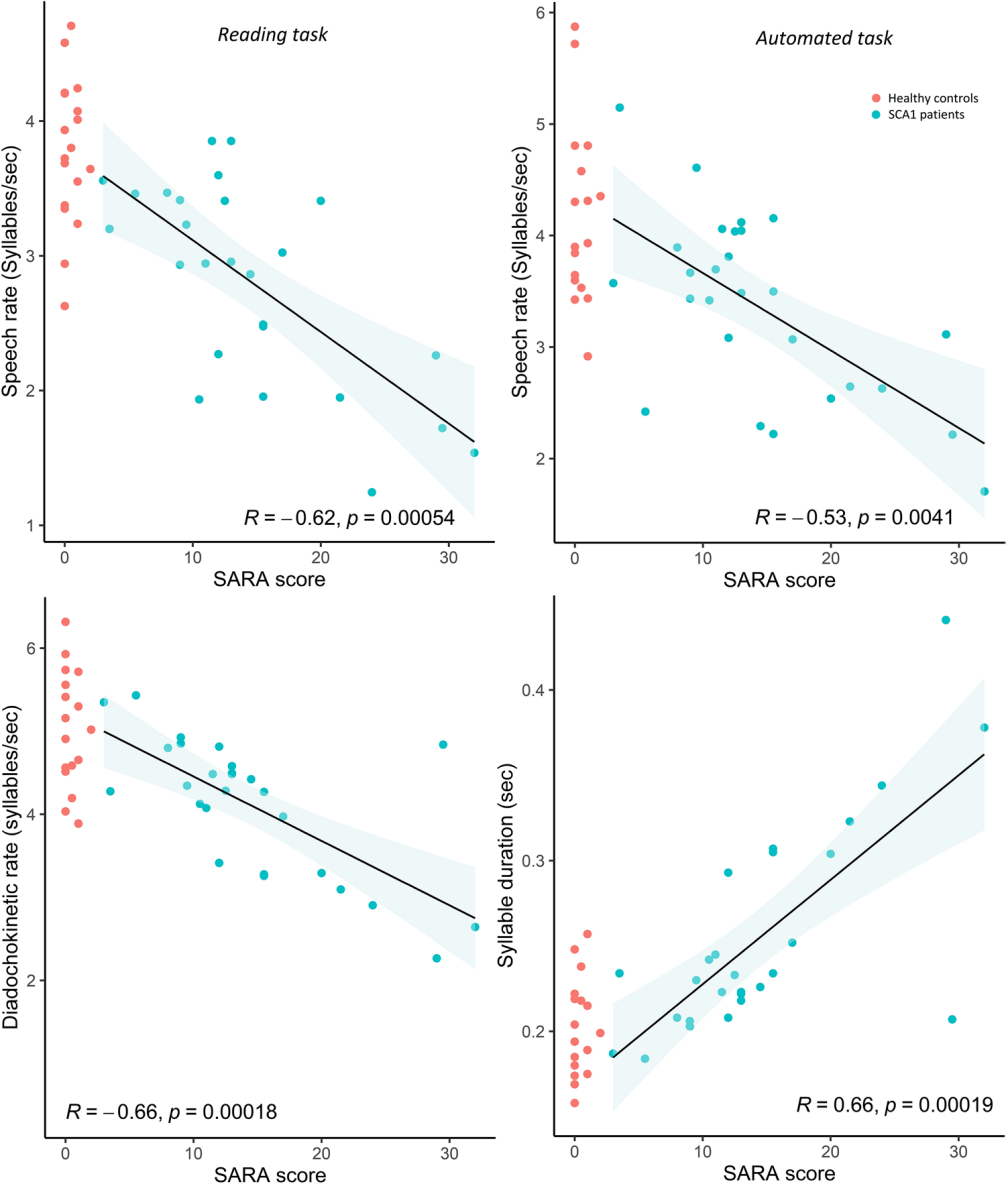
Correlation between acoustic variables (speech rate in reading and automated task, diadochokinetic rate and syllable duration) and disease severity as measured by SARA score

Speech rate (*reading task*: *ρ* = −0.62, *p*-value <0.001 ; “Days of the week” task: *ρ* = −0.53, *p*-value 0.004)) and syllable repetition rate (*ρ* = −0.66, *p*-value <0.001) showed a strong negative association with ataxia severity. Syllable duration (*ρ* = 0.66, *p*-value <0.001) and pause variability in reading task (*ρ* = 0.42, *p*-value 0.03) were positively associated with ataxia severity (see Fig. 2). The correlations between acoustic variables and ataxia severity remained significant with equivalent effect sizes when SARA scores were corrected for speech impairment (correlation with SARA_adj_), reflecting the known tight correlation between the various SARA items (see Appendix 2a). The voice quality measure recurrence period density entropy (*in sustained vowel task*) was not significantly correlated with ataxia severity.

No (Bonferroni-corrected) significant association was observed between acoustic variables and disease duration (see Appendix 2b). However, speech rate *in automated task* (*ρ* = −0.41, *p*-value 0.04) and pause variability *in reading passage* (*ρ* = 0.42, *p*-value 0.04) were significantly correlated with disease duration. We did not observe a significant association between the length of the CAG repeat expansion (see Appendix 2c) in the expanded allele and any acoustic measure of speech. A trend was observed for syllable duration (*ρ* = 0.38, *p*-value 0.05) and syllable repetition rate (*ρ* = −0.38, *p*-value 0.05).

All perceptual measures of speech showed moderate correlation with disease duration (see Appendix 2b). No association between perceptual measures and the length of the CAG repeat expansion was observed.

**Fig. 3:**
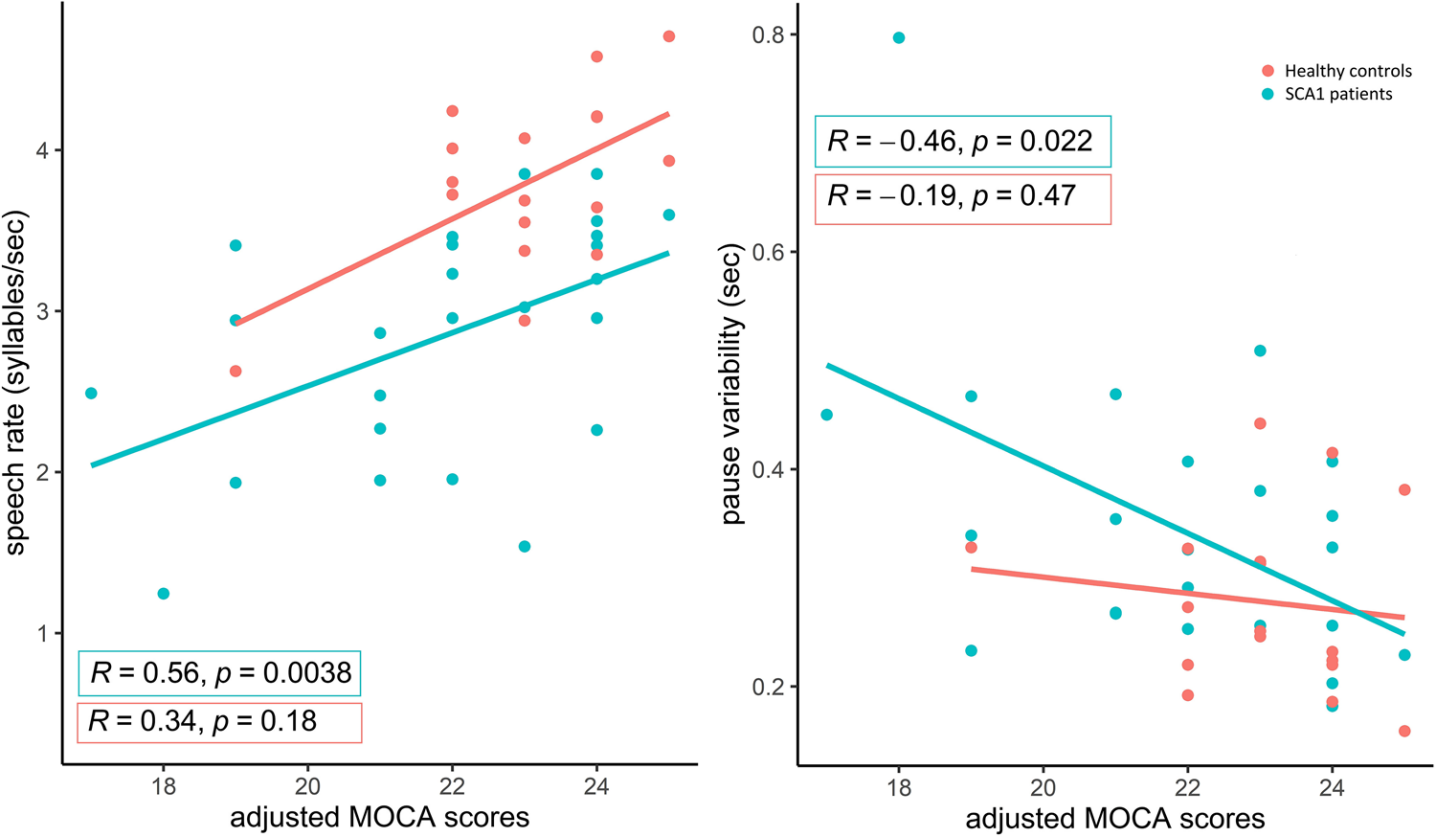
Correlation between adjusted MOCA scores (total score minus visuospatial items) and speech rate and pause variability in reading task for both SCA1 patients and healthy controls

We did find a significant correlation between MoCA_adj_ scores and pause variability *in reading task* (*ρ* = −0.46, *p*-value 0.02), speech rate *in reading task* (*ρ* = 0.56, *p*-value 0.004), syllable duration *task* (*ρ* = −0.46, *p*-value 0.02), and diadochokinetic rate *task* (*ρ* = 0.46, *p*-value 0.02) in SCA1 patients (see Fig. 3)*.* Correlation coefficients for both variables were also calculated for healthy controls, but no significant correlations with MoCA_adj_ or total MoCA scores were observed (see Appendix 2d). Perceptual measures of speech (DME intelligibility: *ρ* = 0.43, *p*-value 0.03, DME naturalness: *ρ* = 0.41, *p*-value 0.04, dysarthria severity score *ρ* = −0.46, *p*-value 0.02) showed a moderate correlation with MoCA_adj_ scores in SCA1 patients.

### SCA1 Patients with No or Minimal Dysarthria vs. Healthy Controls

**Fig. 4:**
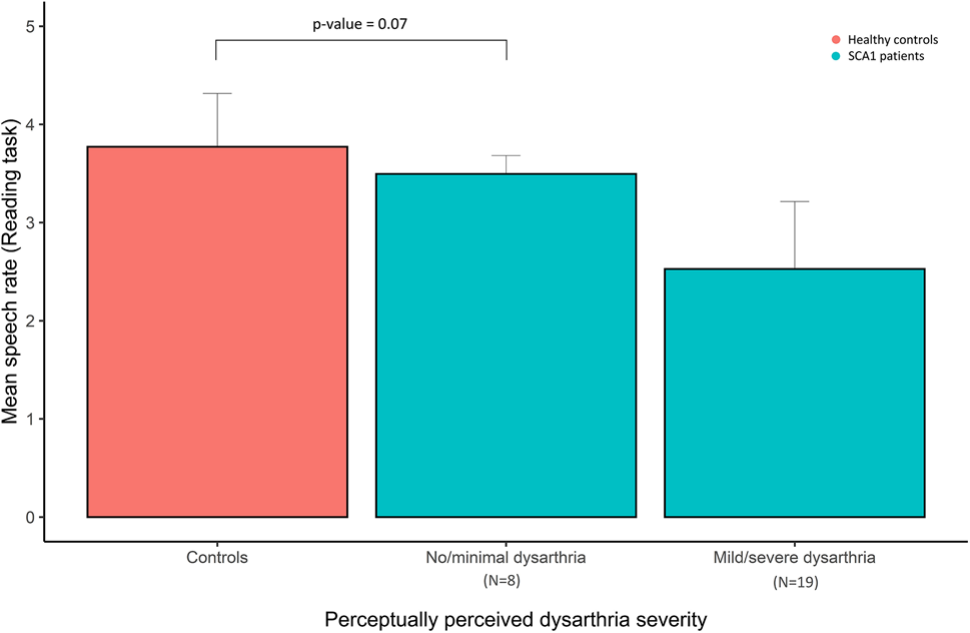
Subgroup analysis for speech rate (reading task) in SCA1 patients with no/minimal dysarthria or mild/severe dysarthria (perceptually rated) vs healthy controls

No statistically significant differences in acoustic variables (speech rate, diadochokinetic rate, syllable duration, and pause variability) were detected between healthy controls and a subgroup of SCA1 patients with subclinical or minimal dysarthria (reflected by dysarthria severity scores: 0 or 1 (*N* = 8)). A trend was observed for speech rate *in reading task* which was lower in SCA1 patients with no or minimal dysarthria compared to healthy controls (*T*-value = −1.93, *p*-value = 0.07) (see Fig. 4).

## Discussion

This study describes perceptual and acoustic speech characteristics of SCA1 patients across symptomatic disease stages. Dysarthria is a common progressive feature in SCA1, as reflected by a strong correlation between all perceptual and several acoustic variables with disease severity in our analysis. The SCA1 speech phenotype is characterized by a slow speech rate and longer syllable duration as well as impaired diadochokinetic rate. The observed speech timing deficits are similar to those observed in other genetic ataxias such as SCA2 [9], FRDA [8, 15], POLG-associated ataxia [11], and ARSCACS [10]. In our cohort, several SCA1 patients showed signs of gait and limb ataxia in the absence of overt dysarthria (both perceptually and acoustically measured), indicating that in some SCA1 patients, gait and limb ataxia precede onset of (detectable) dysarthria. This observation is in line with the previous finding that gait symptoms are the initial manifestation of SCA1 in the majority of patients [26].

Voice quality seemed relatively spared in our cohort, as reflected by the absence of statistically significant between-group differences for acoustic measures. The interpretation of this finding is limited by the absence of a dedicated perceptual analysis of voice quality in this study, which previously revealed abnormalities in SCA1 [6].

No statistically significant subgroup differences were observed in acoustic variables between SCA1 patients with absent of minimal dysarthria (perceptually rated) and healthy controls. We did observe a large variation in the acoustic results (reflected by large standard deviations) within healthy controls, suggesting a potential bias due to sub-maximal performance on acoustic tasks measuring speech rate and diadochokinetic rate. In addition, interpretation of the subgroup analysis is limited by the small sample size of the subgroup with no or minimal dysarthria and the lack of pre-ataxic subjects. The deficits in speech timing previously observed in early and pre-ataxic SCA2 subjects compared to healthy controls [9] were detected in speech samples with higher mean diadochokinetic and speech rates for both SCA2 subjects and healthy control groups compared to these rates in this study. This could suggest that subtle acoustic changes in subclinical dysarthria might be magnified during, and only detectable at, maximal speech performance. An influence of the language difference (Dutch vs. Spanish) between these studies cannot be ruled out.

The cognitive affective cerebellar syndrome (CCAS) is increasingly recognized as a cause of cognitive impairment and language deficits in ataxias [27]. Cognitive dysfunction and impaired phonemic fluency have been previously described in SCA1 [28, 29], but the incidence of CCAS in SCA1 is still unknown. In this study, no significant between-group difference was observed for cognitive functioning between SCA1 patients and healthy controls as measured by MoCA. This finding might be biased by a low mean MoCA score, and remarkably wide range of MoCA scores in healthy controls in this study (mean 27.5 ± 1.5, range 24–30). Additionally, MoCA might not be sensitive to CCAS-related cognitive issues and/or language impairment. Specific cognitive tests, such as the CCAS scale [30], may confirm the presence of cognitive and related language impairment in SCA1. In this study, we did observe a significant correlation between MoCA and both speech timing aspects and perceptual speech measures in SCA1 patients. Additional studies, including CCAS-specific scales, are needed to further address this relationship.

This cross-sectional study has provided baseline speech characteristics of manifest SCA1 mutation carriers across a wide disease span with complementary perceptual and acoustic measurements. Longitudinal data will help clarify whether perceptual and/or acoustic measurements of speech are able to detect changes, and if so, how these compare to other clinical or surrogate markers. We also need to assess how clinician reported and performance outcomes of speech link to specific concepts of interest within the speech domain (e.g., voice quality or speech rate) and whether they reflect meaningful aspects of health for patients [31]. This knowledge is crucial for the design and evaluation of clinical trials that will be initiated in the very near future.

### Supplementary Information

ESM 1
