# Supplementary material for: Perceptual and Acoustic Analysis of Speech in Spinocerebellar ataxia Type 1
Source: Cerebellum. 2023 Jan 12;23(1):112–20. doi: 10.1007/s12311-023-01513-9 (PMC10864471; doi:10.1007/s12311-023-01513-9)
Supplement: Supplementary file 1 — ESM 1 [file 12311_2023_1513_MOESM1_ESM.docx]

| Appendix 1a. Mean, SD, Mann-Whitney U test results and Effect size for all perceptual variables in SCA1 mutation carriers and healthy controls | | | | | | | | | |
| --- | --- | --- | --- | --- | --- | --- | --- | --- | --- |
|  | | **SCA1 mutation carriers** | | | **Healthy controls** | | | **Mann-Whitney U** | **Effect size** |
| Perceptual variables | **Rated in:** | *n* | *Mean* | *SD* | *n* | *Mean* | *SD* | *p-value* | *Cohen's D* |
| DME intelligibility | Monologue | 27 | 102,0 | 54,8 | 18 | 194,4 | 23,6 | **<0.0001** | -2,19 |
| DME naturalness | Monologue | 27 | 96,3 | 59,5 | 18 | 194,4 | 23,6 | **<0.0001** | -2,17 |
| Dysarthria severity score | Monologue | 27 | 2,2 | 1,4 | 18 | 0,11 | 0,3 | **<0.0001** | 2,03 |

| Appendix 1b. Mean, SD, T-test results and Effect size for all acoustic variables in SCA1 mutation carriers and healthy controls | | | | | | | | | | |
| --- | --- | --- | --- | --- | --- | --- | --- | --- | --- | --- |
|  | | **SCA1 mutation carriers** | | | **Healthy controls** | | | **T-test** | | **Effect size** |
| Acoustic variables | **Stimuli extracted from:** | *n* | *Mean* | *SD* | *n* | *Mean* | *SD* | *T-value* | *p-value* | *Cohen's D* |
| Fundamental frequency coefficient of variation | sustained vowel | 26 | 0,03 | 0,02 | 18 | 0,03 | 0,03 | 1.43 | 0.16 | 0,18 |
| Harmonics to Noise Ratio (mean) | sustained vowel | 27 | 22,8 | 4,32 | 18 | 25,59 | 5,03 | -1.9 | 0.07 | -0,59 |
| Recurrence Period Density Entropy | sustained vowel | 27 | 0,6 | 0,08 | 18 | 0,57 | 0,09 | 2.23 | 0.03 | 0,69 |
| Speech rate (syllable/second) | Days of the week | 27 | 3,4 | 0,82 | 17 | 4,18 | 0,80 | -3.28 | **0.002** | -1,01 |
| Fundamental frequency coefficient of variation | Monologue | 27 | 0,20 | 0,06 | 18 | 0,19 | 0,04 | 0.22 | 0.82 | 0,10 |
| Pause length (mean) | Monologue | 27 | 0,29 | 0,19 | 18 | 0,26 | 0,11 | 0.51 | 0.61 | 0,19 |
| Percent of pauses (%) | Monologue | 27 | 25,0 | 9,31 | 18 | 21,5 | 6,47 | 1.46 | 0.15 | 0,44 |
| Syllable duration (seconds) | Syllable repetition task (PA-TA-KA) | 27 | 0,26 | 0,06 | 18 | 0,21 | 0,03 | 3.65 | **<0.001** | 1,00 |
| Syllable repetition rate (syllable/second) | Syllable repetition task (PA-TA-KA) | 27 | 4,11 | 0,82 | 18 | 4,97 | 0,73 | -3.67 | **<0.001** | -1,10 |
| Syllable variability | Syllable repetition task (PA-TA-KA) | 27 | 0,31 | 0,10 | 18 | 0,32 | 0,08 | -0.61 | 0.55 | -0,12 |
| Pause length (mean) | North wind and Sun (read passage) | 27 | 0,31 | 0,12 | 18 | 0,25 | 0,08 | 1.96 | 0.06 | 0,58 |
| Variability in pause length | North wind and Sun (read passage) | 27 | 0,36 | 0,14 | 18 | 0,28 | 0,08 | 2.45 | 0.02 | 0,71 |
| Speech rate (syllable/second) | North wind and Sun (read passage) | 27 | 2,81 | 0,74 | 18 | 3,77 | 0,54 | -5.03 | **<0.0001** | -1,48 |

*Note: The following acoustic variables were log natural transformed (did not present with gaussian distribution) before statistical testing:* *Fundamental frequency coefficient of variation in sustained vowel and monologue, pause length in reading task and monologue, pause variability in reading task, percent of pauses in monologue, syllable duration and syllable variability in syllable repetition task.*

*p-values < 0.05 in bold.*

| Appendix 2a. Correlation between SARA score (and SARA­adjusted) and relevant acoustic and perceptual variables in SCA1 mutation carriers | | | | | |
| --- | --- | --- | --- | --- | --- |
|  | | **SARA score** | | **Adjusted SARA score** | |
|  | | **Correlation coefficient**  **(Spearman Rho)** | **p-value** | **Correlation coefficient**  **(Spearman Rho)** | **p-value** |
| Acoustic variables | **Rated in:** |  |  |  |  |
| Speech rate (syllable/second) | North wind and Sun (read passage) | -0.62 | **<0.001** | -0.6 | **<0.001** |
| Variability in pause length | North wind and Sun (read passage) | 0.42 | **0.03** | 0.42 | **0.03** |
| Syllable duration (seconds) | Syllable repetition task (PA-TA-KA) | 0.66 | **<0.001** | 0.66 | **<0.001** |
| Syllable repetition rate (syllable/second) | Syllable repetition task (PA-TA-KA) | -0.66 | **<0.001** | -0.66 | **<0.001** |
| Speech rate (syllable/second) | Days of the week | -0.53 | **0.004** | -0.51 | **0.007** |
| Recurrence Period Density Entropy (mean) | sustained vowel | 0.25 | 0.21 | 0.2 | 0.33 |
|  |  |  |  |  |  |
| Perceptual variables | **Rated in:** |  |  |  |  |
| DME intelligibility | Monologue | -0.74 | **<0.0001** | -0.7 | **<0.0001** |
| DME naturalness | Monologue | -0.81 | **<0.0001** | -0.78 | **<0.0001** |
| Dysarthria severity scale | Monologue | 0.78 | **<0.0001** | 0.74 | **<0.0001** |

| Appendix 2b. Correlation between disease duration and relevant acoustic and perceptual variables in SCA1 mutation carriers | | | |
| --- | --- | --- | --- |
|  | | **Disease duration** | |
|  | | **Correlation coefficient**  **(Spearman Rho)** | **p-value** |
| Acoustic variables | **Rated in:** |  |  |
| Speech rate (syllable/second) | North wind and Sun (read passage) | -0.29 | 0.16 |
| Variability in pause length | North wind and Sun (read passage) | 0.42 | **0.04** |
| Syllable duration (seconds) | Syllable repetition task (PA-TA-KA) | 0.22 | 0.29 |
| Syllable repetition rate (syllable/second) | Syllable repetition task (PA-TA-KA) | -0.23 | 0.28 |
| Speech rate (syllable/second) | Days of the week | -0.41 | **0.04** |
| Recurrence Period Density Entropy (mean) | sustained vowel | 0.23 | 0.27 |
|  |  |  |  |
| Perceptual variables | **Rated in:** |  |  |
| DME intelligibility | Monologue | 0.40 | 0.06 |
| DME naturalness | Monologue | -0.42 | **0.04** |
| Dysarthria severity score | Monologue | 0.46 | **0.03** |

| Appendix 2c. Correlation between expanded CAG repeat and relevant acoustic and perceptual variables in SCA1 patients | | | |
| --- | --- | --- | --- |
|  | | **Correlation coefficient**  **(Spearman Rho)** | **p-value** |
| Acoustic variables | **Rated in:** |  |  |
| Speech rate (syllable/second) | North wind and Sun (read passage) | -0.34 | 0.09 |
| Variability in pause length | North wind and Sun (read passage) | 0.04 | 0.86 |
| Syllable duration (seconds) | Syllable repetition task (PA-TA-KA) | 0.38 | 0.05 |
| Syllable repetition rate (syllable/second) | Syllable repetition task (PA-TA-KA) | -0.38 | 0.05 |
| Speech rate (syllable/second) | Days of the week | -0.07 | 0.75 |
| Recurrence Period Density Entropy (mean) | sustained vowel | 0.12 | 0.56 |
|  |  |  |  |
| Perceptual variables | **Rated in:** |  |  |
| DME intelligibility | Monologue | -0.11 | 0.59 |
| DME naturalness | Monologue | -0.2 | 0.33 |
| Dysarthria severity score | Monologue | 0.18 | 0.38 |

| Appendix 2d. Correlation between cognitive functioning (adjusted MOCA scores) and relevant acoustic and perceptual variables in SCA1 patients and healthy controls | | | | | |
| --- | --- | --- | --- | --- | --- |
|  | | **SCA1 patients** | | **Healthy controls** | |
|  |  | **Correlation coefficient**  **(Spearman Rho)** | **p-value** | **Correlation coefficient**  **(Spearman Rho)** | **p-value** |
| Acoustic variables | **Rated in:** |  |  |  |  |
| Speech rate (syllable/second) | North wind and Sun (read passage) | 0.56 | **0.004** | 0.34 | 0.17 |
| Variability in pause length | North wind and Sun (read passage) | -0.46 | **0.02** | -0.19 | 0.46 |
| Syllable duration (seconds) | Syllable repetition task (PA-TA-KA) | -0.46 | **0.02** | -0.30 | 0.23 |
| Syllable repetition rate (syllable/second) | Syllable repetition task (PA-TA-KA) | 0.46 | **0.02** | 0.30 | 0.24 |
| Speech rate (syllable/second) | Days of the week | 0.36 | 0.08 | 0.13 | 0.63 |
| Recurrence Period Density Entropy (mean) | sustained vowel | -0.12 | 0.57 | -0.06 | 0.80 |
|  |  |  |  |  |  |
| Perceptual variables | **Rated in:** |  |  |  |  |
| DME intelligibility | Monologue | 0.43 | **0.03** | 0.02 | 0.92 |
| DME naturalness | Monologue | 0.41 | **0.04** | 0.02 | 0.92 |
| Dysarthria severity score | Monologue | -0.46 | **0.02** | 0.14 | 0.58 |
